# Risk of childhood cerebral palsy following prenatal exposure to ß2-adrenergic receptor agonist: A nationwide cohort study

**DOI:** 10.1371/journal.pone.0202078

**Published:** 2018-08-16

**Authors:** Lin Li, Ziliang Wang, Hong Liang, Fen Yang, Wei Yuan, Bizu Gelaye, Yongfu Yu, Maohua Miao, Mette Nørgaard, Jiong Li

**Affiliations:** 1 Key Lab. of Reproduction Regulation of NPFPC, SIPPR, IRD, Fudan University, Shanghai, China; 2 Department of Clinical Epidemiology, Aarhus University Hospital, Aarhus, Denmark; 3 School of Public Health, Fudan University, Shanghai, China; 4 Department of Epidemiology, Harvard T. H. Chan School of Public Health, Boston, United States of America; Amsterdam UMC, location AMC, NETHERLANDS

## Abstract

**Background:**

Cerebral palsy (CP) is the most common physical developmental disability in childhood with a prevalence of 2 to 3 per 1000 live births. β2-adrenoreceptor agonist (β2AA) are widely used for the treatment of asthma. Maternal use of β2AAs may increase the risk of adverse neuro-psychiatric health outcomes in the offspring. No study, however, has evaluated the effect of prenatal exposure to β2AAs on the risk of CP.

**Objective:**

To examine the association between prenatal exposure to β2AAs and the risk of childhood cerebral palsy.

**Methods:**

This population-based cohort study included all live singleton births in Denmark from January 1, 1997 to December 31, 2003. The information on outpatient prescriptions of β2AAs was extracted from Danish National Prescription Registry. Children born to mothers who used β2AAs from 30 days before pregnancy until delivery were categorized as the exposed. To differentiate the effect of β2AAs from the underlying indications, the exposure window was further extended to 2 years before pregnancy and the exposed groups were re-defined to represent different periods of exposure to maternal use of β2AAs (use only before pregnancy, use only during pregnancy, and use both before and during pregnancy). Cases of CP were identified from the Danish Cerebral Palsy Register. Logistic regression was used to estimate incidence odds ratio (OR) of CP.

**Results:**

Among all the 442,278 singletons, 19,616 (4.44%) were exposed to β2AAs in utero (from 30 days before pregnancy until delivery). The risk of childhood CP was 0.21% in exposed and 0.19% in unexposed group, yielding an adjusted OR (aOR) 1.12 (95% confidence interval (CI): 0.82, 1.53). When extending the exposure time window to 2 years prior to pregnancy, no overall significant association was observed regardless of the exposure period. However, an increased risk of CP (aOR = 1.41, 95%CI: 0.92, 2.18) for maternal β2AAs use during pregnancy was observed in female offspring, especially in those born at term (aOR = 1.65, 95%CI: 1.02, 2.67). This increase was mainly attributed to an increased risk in those born to mothers who used β2AAs both before and during pregnancy (aOR = 1.81, 95%CI: 0.99, 3.33).

**Conclusions:**

We observed an association between maternal β2AAs use during pregnancy and an increased risk of CP in female offspring, but we could not rule out confounding by the underlying indications for β2AAs.

## Introduction

Cerebral palsy (CP) represents a range of non-progressive syndromes of posture and motor impairment that result from an insult to the developing central nervous system (CNS). It is the most common physical developmental disability in childhood with a prevalence of 2 to 3 per 1000 live births, causing limitation of activity to varied degrees as well as disturbance of sensation, cognition, communication, perception, and behavior [1–5].CP imposes severe demands on health, educational and social services, as well as on the families and children themselves [1, 6].

The etiology of the majority of CP cases remains unexplained [7]. In addition to adverse birth outcomes (preterm birth, low birth weight, and low 5-minute Apgar score) [8, 9], several prenatal factors, such as prenatal stress[10], maternal infection and smoking [11], and prenatal exposure to perfluoroalkyl substances (PFASs) [12], have been shown to be associated with CP. This suggests that maternal in-utero environment may be important for the development of CP in the offspring. As pregnant women are most often excluded from clinical trials [13], there is very limited information about the effects of drugs prescribed during pregnancy on the risk of offspring’s CP [14].

ß2-adrenergic receptor agonists (β2AAs) are widely used for the treatment of asthma that affects up to 8% of pregnancies [15]. Women having asthma are recommended to continue their medication during pregnancy with a few exceptions since uncontrolled asthma may lead to a number of adverse birth outcomes [16]. ß2AAs have also been off-label used for management of preterm labor as tocolytics. ß-2-adrenergic receptors are highly expressed during fetal development and the receptors are readily capable of modulating intracellular cAMP production [17]. β2AAs can cross placenta and blood-brain barrier and stimulate ß2-adrenergic receptors in all tissues of the fetus [18, 19]. Thus, exposure during pregnancy may increase fetal ß2-adrenergic receptors signaling or overstimulate the fetal ß2-adrenergic receptor and may act as functional and behavioral teratogens and permanently alter the balance of sympathetic and parasympathetic tone, resulting in neurodevelopmental disorders. [20, 21]

Long term use of terbutaline for prevention of preterm birth during the second or third trimester has been associated with an increased risk of Autism Spectrum Disorders (ASD) [22, 23]. A previous Danish case-control study similarly suggested that any β2AA exposure during pregnancy or preconception may be associated with an increased risk of ASD [24]. However, human studies on neurodevelopmental effects of β2AAs are scant and with small sample sizes. Furthermore, no study has evaluated the effect of prenatal exposure to β2AAs on the risk of CP. ß-2-adrenergic receptors can influence regulation of vascular tone, which would alter blood flow to and within the brain and placenta. This can change the responsiveness of the vasculature to infection, pro-coagulant state, hypoxia, or hyperoxia [25, 26]. Therefore, we hypothesized that maternal β2AAs use during pregnancy would be associated with the risk of CP in offspring. In this large population-based study, we aimed to examine the association between maternal β2AAs use and the risk of CP in the offspring. As animal studies have suggested that prenatal β2AAs exposure caused sex-related effects on macromolecules and DNA synthesis in the immature brain [27], we also speculated there might exist a sex-specific effect of β2AAs in humans.

## Materials and methods

### Study population and design

This cohort study was based on data from several Danish national registers. Linkage between the registers was achieved via the unique personal registration number (CPR number), which is assigned to all citizens in Denmark and used in all nationwide registries [28]. The CPR number enabled accurate linkage of all registries at the individual level [28]. We identified all live singletons born in Denmark between January 1, 1997 and December 31, 2003 from the Danish Medical Birth Register (DMBR), which has included all births in Denmark since 1973 [29]. A total of 442,690 births were recorded during the study period. We excluded 15 infants with missing information on maternal parity and 397 infants whose gestational age at birth was ≤23 weeks or ≥45 weeks. A total of 442,278 singletons were eligible for the study. All the children were followed from birth until first diagnosis of CP, death, emigration, or the end of 2003, whichever came first.

### Maternal use of β2AAs

We obtained the information on outpatient prescriptions of β2AAs and dispensing date from Danish National Prescription Registry (DNPR), which covers individual-level information on all prescriptions dispensed at Danish outpatient pharmacies since 1995, except hospital dispensaries in Denmark [30].We extracted prescriptions for β2AAs using the Anatomical Therapeutic Chemical (ATC) code of R03AC or R03CC. Exposure during a given period was defined as the dispensing date fell within the specified period or the number of days for which the β2AAs was supplied overlapped with the given period. We defined exposed children as those born to mothers who redeemed a prescription for β2AAs from 30 days before pregnancy until delivery (use during pregnancy), while unexposed children were those born to mother who did not redeem any prescription of β2AAs during the same period (no use during pregnancy). For further analyses, we also extended the exposure window and obtained data on redemption of a β2AA prescription 2 years before pregnancy until delivery (detailed in Statistical Analyses). We estimated the cumulative dose of β2AAs by first multiplying number of packages redeemed for each prescription and number of defined daily doses in each package, and then summing all prescriptions within the exposure window. We categorized the cumulative dose into high and low levels based on the 75^th^ percentile. In addition, we specifically examined two main subgroups of β2AAs: salbutamol, and terbutaline.

### Outcome

Information on CP diagnosis was identified from the Danish Cerebral Palsy Register [6]. The register contains all individuals who survived to at least 1 year of age with a diagnosis of CP validated by a neuro-pediatrician in Denmark since 1995 [8, 31]. The definition of CP used in the register was from Surveillance of Cerebral Palsy in Europe [1], as a group of permanent movement and/or posture disorders caused by nonprogressive interference/lesion/abnormality in the developing/immature brain.

### Covariates

Information on year of birth, gender, birth weight, gestational age, 5-minute Apgar score, parental age at delivery, maternal parity, and maternal smoking during pregnancy were retrieved from DMBR [32]. Information on maternal country of origin was obtained from the Danish Civil Registration System [33]. We obtained information on maternal sociodemographic status and maternal education level from Integrated Database for longitudinal Labor Market Research [34]. Information on family history of CP (ICD 8 codes:343–344 from 1977–1993, ICD 10 codes:G80-G83 from 1994 onward) and maternal history of hospital-diagnosed asthma before delivery (ICD8 codes: 493, ICD10 codes: J45-J46) were extracted from Danish National Patient Register [35]. Asthma is often treated in primary care, and hospital-diagnosed asthma may be seen as an indicator of more severe asthma. In addition, information on maternal use of inhaled glucocorticoids (ATC: R03BA) during pregnancy were extracted from DNPR, since combinations of β2AAs and inhaled glucocorticoids were recommended for asthma treatment according to clinical guidelines [36].

### Statistical analyses

All analyses were performed using SAS version 9.1 (SAS Institute Inc,Cary, North Carolina, USA). Logistic regression model was used to estimate odds ratios (ORs) and corresponding 95% confidence intervals (CIs) of CP in offspring in relation to maternal use of β2AAs during pregnancy.

We performed two statistical models to estimate adjusted ORs. In model 1, we adjusted for potential confounders that were previously described as risk factors of CP or factors associated with β2AAs use, including child’s gender (male, female), year of birth (1997–1998, 1999–2000, 2001–2002, 2003), parity (1, 2, 3), maternal age at delivery (≤25, 26–30, >30 years), paternal age (≤25, 26–30,>30 years, unknown), maternal education (≤9, 10–14, ≥15 years), maternal cohabitation status (single, cohabitation, unknown), maternal smoking status (yes, no), and maternal history of CP (yes, no). In addition, other potential confounders, such as maternal country of origin, maternal employment status, and paternal history of CP, were evaluated but not included in model 1 because they changed effect estimates of interest by less than 1%. In model 2, we additionally adjusted for maternal asthma hospital-diagnosed before delivery to control for potential confounding by severity of the main indication. We did not adjust for 5-minute Apgar score and birth weight, as they might be on the causal pathway between maternal β2AAs use during pregnancy and CP in offspring, or share the same risk factors with CP.

Animal studies suggested that prenatal β2AAs exposure caused sex-related effects on macromolecules and DNA synthesis in immature brain [27], therefore, we stratified analyses by gender to examine whether children’s gender modified the association between maternal β2AAs use and CP. In addition, our exposure data were restricted to only outpatient prescriptions. Thus we did not have information on in-hospital use of β2AAs as a tocolytic agent, which means that children exposed to maternal β2AAs use for preterm pregnancies were actually misclassified as unexposed. Therefore, we further restricted analyses to children born at term, too.

To differentiate the effect of β2AAs from the underlying indications, we further extended the exposure window to the time period from 2 years before pregnancy to delivery and redefined exposed groups as children born to mothers who redeemed β2AAs medication: 1) only from 2 years before pregnancy until 30 days before pregnancy (use only before pregnancy); 2) only during the period from 30 days before pregnancy until delivery (use only during pregnancy); 3) both during the period from 2 years before pregnancy until 30 days before pregnancy and during the period from 30 days prior to pregnancy until delivery (use both before and during pregnancy). The reference group consisted of children whose mothers did not redeem β2AAs during the period from 2 years before pregnancy until delivery (never use). We compared the risks of CP in offspring related to maternal β2AAs use during different exposure periods. We hypothesized that it would be more likely an effect of indication rather than medication if β2AAs use only during pregnancy was associated with a similar risk of CP as the use only before pregnancy.

For children included in the Danish CP register before the age of 4 years, a new report will be required at the age of 4–5 years to ensure that the diagnosis is correctly evaluated [6]. This study included children born up to the end of follow-up of 2003, thus CP cases born after 2000 will not have a chance for receiving a validated CP diagnosis. To examine the potential bias, we further restricted our main analyses to children born before 1999, who were at least 5 years old by the end of 2003. Other subanalyses included: estimation of ORs for CP after exposure to high and low dose levels of β2AAs, and ORs for CP after exposure to specific β2AAs, salbutamol and terbutaline. In addition, to exclude the effect of co-medication, we stratified the main analyses by maternal use of glucocorticoids.

### Ethic statement

According to Danish law, register-based studies do not require consent from individuals when personal identifiers are encrypted and stored by a trusted third party (Statistic Denmark). This study was approved by the Danish Data Protection Agency (No. 2013-41-2569).

## Results

We identified 442,278 singletons in the cohort, of whom 19,616 (4.44%) were exposed to β2AAs in utero. **Table 1** shows the baseline characteristics of exposed and unexposed groups. Exposed children were born to mothers having more previous deliveries, being less educated, being outside of the labor market, smoking more, and having a higher prevalence of asthma. In addition, exposed children tended to be born in earlier calendar years.

**Table 1:** Baseline characteristics of the study population according to β2AAs usage during pregnancy.

Characteristic	β2AAs exposure during pregnancy (N = 19616)	No β2AAs exposure during pregnancy (N = 422662)
	N (%)	N (%)
**Year of birth**		
1997–1998	6583(33.56)	122045(28.88)
1999–2000	5514(28.11)	122466(28.97)
2001–2002	5156(26.28)	118686(28.08)
2003	2363(12.05)	59465(14.07)
**Gender**		
Male	10064(51.31)	216934(51.33)
Female	9552(48.69)	205728(48.67)
**Birth Weight (g)**		
<2500	853(4.35)	14643(3.46)
2500–3250	4957(25.27)	98452(23.29)
3250–4000	9817(50.05)	219546(51.94)
4000–8000	3812(19.43)	86011(20.35)
Missing	177(0.90)	4010(0.95)
**Parity**		
1	7221(36.81)	182344(43.14)
2	7829(39.91)	158348(37.46)
3	4566(23.28)	81970(19.39)
**Preterm Birth (<37 weeks)**		
No	18514(94.38)	402578(95.25)
Yes	1102(5.62)	20084(4.75)
**Apgar score at 5 minutes**		
0–7	293(1.49)	5674(1.34)
8–9	1236(6.30)	25711(6.08)
10	17831(90.90)	385480(91.20)
Missing	256(1.31)	5797(1.37)
**Maternal age (years)**		
≤25	3603(18.37)	73638(17.42)
26–30	7141(36.40)	160668(38.01)
>30	8872(45.23)	188356(44.56)
**Paternal age (years)**		
≤25	1822(9.29)	33078(7.83)
26–30	5348(27.26)	119602(28.30)
>30	11877(60.55)	259914(61.49)
Missing	569(2.90)	10068(2.38)
**Maternal education (years)**		
≤9	5564(28.36)	92039(21.78)
10–14	9145(46.62)	210228(49.74)
≥15	4554(23.22)	108976(25.78)
Missing	353(1.80)	11419(2.70)
**Maternal smoking status**		
Yes	5248(26.75)	82703(19.57)
No	13468(68.86)	321220(76.00)
Missing	900(4.59)	18739(4.43)
**Maternal employment status**		
Outside labor market	5718(29.15)	104602(24.75)
Blue collar workers	1336(6.81)	29679(7.02)
White collar workers	7384(37.64)	161456(38.20)
Top level status	5114(26.07)	122932(29.90)
Missing	64(0.33)	3993(0.94)
**Maternal country of birth**		
Nordic	19425(99.03)	418947(99.12)
Others	190(0.97)	3678(0.87)
Missing	1(0.01)	37(0.01)
**Maternal cohabitation status**		
Single	10026(51.11)	203860(48.23)
Cohabitation	9528(48.57)	214869(50.84)
Missing	62(0.32)	3933(0.93)
**Maternal history of cerebral palsy**		
No	19552(99.67)	421910(99.82)
Yes	64(0.33)	752(0.18)
**Paternal history of cerebral palsy**		
No	19576(99.80)	421907(99.82)
Yes	40(0.2)	755(0.18)
**Maternal history of asthma hospital-diagnosed**		
No	15635(79.71)	418047(98.91)
Yes	3981(20.29)	4615(1.09)

A total of 843 children (0.19%) were diagnosed with CP with median age of 0.96 (0.54, 1.91) years at diagnosis. The prevalence of childhood CP was 0.21% in exposed group and 0.19% in unexposed group. No association between maternal use of β2AAs during pregnancy and the risk of CP in the offspring was observed after adjusting for potential confounders. When we extended the exposure window to 2 years prior to pregnancy and redefined the exposed and unexposed groups, no overall significant association was observed regardless of the exposure period **(Table 2)**.

**Table 2:** Association between maternal β2AAs usage and cerebral palsy in offspring.

Beta 2 adrenoreceptor agonists use	Offspring without CP (n = 441,435)	Offspring with CP (n = 843)	cOR(95%CI)	Model 1 aOR(95%CI)^a^	Model 2 aOR(95%CI)^b^
**No use during pregnancy**	421861(99.81)	801(0.19)	Ref	Ref	Ref
**Use during pregnancy**	19574(99.79)	42(0.21)	1.13(0.83,1.54)	1.12(0.82,1.53)	1.10(0.78,1.53)
**Never use**	389416 (99.81)	738(0.19)	Ref	Ref	Ref
**Use only before pregnancy**	32445(99.81)	63(0.19)	1.03(0.79,1.33)	1.07(0.82,1.39)	1.06(0.82,1.39)
**Use only during pregnancy**	8390(99.82)	15(0.18)	0.94(0.57,1.57)	0.94(0.57,1.58)	0.94(0.56,1.58)
**Use both before and during pregnancy**	11184(99.76)	27(0.24)	1.27(0.87,1.87)	1.26(0.86,1.85)	1.26(0.83,1.90)

^a^Adjusted for year of birth, gender, parity, maternal age, paternal age, maternal cohabitation status, maternal education, maternal smoking, maternal history of cerebral palsy.

^b^Additionally adjusted for maternal history of hospital–diagnosed asthma based on model 1.

When examining male and female offspring separately we found, as shown in **Table 3**, that there was a higher but non-statistically significant risk of CP in female offspring (aOR = 1.41, 95%CI: 0.92, 2.18) (Model 1). This increased risk was mainly attributed to an increased risk in those born to mothers who used β2AAs both before and during pregnancy (aOR = 1.60, 95%CI: 0.94, 2.75) (Model 1). However, no similar patterns were observed in male offspring.

**Table 3:** Association between maternal β2AAs usage and cerebral palsy in offspring by gender.

Beta 2 adrenoreceptor agonists use	Offspring without CP	Offspring with CP	cOR(95%CI)	Model 1 aOR(95%CI)^a^	Model 2 aOR(95%CI)^b^
***MALE***	**n = 226501**	**n = 497**			
**No use during pregnancy**	216457(99.78)	477(0.22)	Ref	Ref	Ref
**Use during pregnancy**	10044(99.80)	20(0.20)	0.90(0.58,1.41)	0.91(0.58,1.42)	0.89(0.56,1.43)
**Never use**	199728(99.78)	441(0.22)	Ref	Ref	
**Use only before pregnancy**	16729(99.79)	36 (0.21)	0.98 (0.69,1.37)	1.03(0.73,1.47)	1.04(0.73,1.47)
**Use only during pregnancy**	4261(99.84)	7(0.16)	0.75(0.35,1.57)	0.76(0.36,1.60)	0.76(0.34,1.60)
**Use both before and during pregnancy**	5783(99.78)	13(0.22)	1.02(0.59,1.77)	1.02(0.59,1.78)	1.01(0.56,1.83)
***FEMALE***	**N = 214934**	**N = 346**			
**No use during pregnancy**	205404(99.84)	324(0.16)	Ref	Ref	Ref
**Use during pregnancy**	9530(99.76)	22(0.24)	1.46(0.95,2.26)	1.41(0.92,2.18)	1.41(0.89,2.22)
**Never use**	189688(99.84)	297(0.16)	Ref	Ref	
**Use only before pregnancy**	15716(99.83)	27(0.17)	1.10(0.74,1.63)	1.11(0.74,1.66)	1.11(0.74,1.67)
**Use only during pregnancy**	4129(99.81)	8(0.19)	1.24(0.61,2.50)	1.20(0.59,2.42)	1.20(0.59,2.43)
**Use both before and during pregnancy**	5401(99.74)	14(0.26)	1.66(0.97,2.83)	1.60(0.94,2.75)	1.62(0.91,2.88)

^a^Adjusted for year of birth, parity, maternal age, paternal age, maternal cohabitation status, maternal education, maternal smoking, maternal history of cerebral palsy.

^b^Additionally adjusted for maternal history of hospital–diagnosed asthma based on model 1.

Restricting to term births, **Table 4** shows an association between maternal use of β2AAs during pregnancy and an increased risk of CP in female offspring born at term (aOR = 1.65, 95%CI: 1.02, 2.67) (Model 1). This increased risk was mainly attributed to an increased risk in the offspring born to mothers who used β2AAs both before and during pregnancy, which was statistically significant after further adjustment for maternal hospital-diagnosed asthma (aOR = 2.05, 95%CI: 1.08, 3.88) (Model 2), although a non-significant higher risk was also found in those born to mothers who used β2AAs only during pregnancy (aOR = 1.52, 95%CI: 0.72, 3.24) (Model 1).

**Table 4:** Association between maternal β2AAs usage and cerebral palsy in female offspring born at term.

Beta 2 adrenoreceptor agonists use	Offspring without CP (n = 205,503)	Offspring with CP (n = 250)	cOR(95%CI)	Model 1 aOR(95%CI)^a^	Model 2 aOR(95%CI)^b^
**No use during pregnancy**	196453(99.88)	232(0.12)	Ref	Ref	Ref
**Use during pregnancy**	9050(99.80)	18(0.20)	1.68(1.04,2.72)*	1.65(1.02,2.67)*	1.77(1.07,2.91)*
**Never use**	181413(99.88)	210(0.12)	Ref	Ref	
**Use only before pregnancy**	15040(99.85)	22(0.15)	1.26(0.81,1.96)	1.26(0.80,1.98)	1.29(0.82,2.00)
**Use only during pregnancy**	3903(99.82)	7(0.18)	1.55(0.73,3.29)	1.52(0.72,3.24)	1.57(0.74,3.45)
**Use both before and during pregnancy**	5147(99.79)	11(0.21)	1.85(1.01,3.39)*	1.81(0.99,3.33)	2.05(1.08,3.88)*

^a^Adjusted for year of birth, parity, maternal age, paternal age, maternal cohabitation status, maternal education, maternal smoking, maternal history of cerebral palsy.

^b^Additionally adjusted for maternal history of hospital–diagnosed asthma based on model 1.

*p<0.05.

Subanalyses in children born before 1999 showed a stronger association between maternal use of β2AAs during pregnancy and an increased risk of CP in female offspring born at term (aOR = 2.34, 95%CI: 1.16, 4.69) (**S1 Table**) Similarly, the increased risk was mainly attributed to maternal β2AAs use both before and during pregnancy (aOR = 2.86, 95%CI: 1.22, 6.71). An increased risk of CP was associated with a low dose level of β2AAs, but we did not observed a dose-response association (**S2 Table**). In addition, increased risks of CP were observed when we specifically examined salbutamol and terbutaline, although only effect of salbutamol reached statistical significance (**S3 Table**). Finally, when we restricted the main analyses in subgroup with no maternal use of glucocorticoids, there was a similar trend of increased CP risk in female offspring born at term who were exposed to maternal use of β2AAs, although it did not reach statistical significance (**S4 Table**). The results of the subgroup with glucocorticoids use might not be informative due to the limited number of cases (data not shown).

## Discussion

To our knowledge, this is the first large population-based cohort study to explore the association between prenatal exposure to maternal β2AAs and risk of childhood cerebral palsy. We observed an association between maternal use of β2AAs during pregnancy and an increased risk of CP in female offspring born at term.

Our findings are consistent with previous studies, which reported prenatal exposure to the β2AAs has been associated with poor school performance [37], lower cognitive performance and motor development, and increased risk of neuro-psychiatric disorders in children [38].

There are mainly two plausible causes that might lead to the increased risk of CP in offspring exposed to maternal use of β2AAs: β2AAs *per se* and the underlying diseases indicating β2AAs use. Rodent studies suggest that prenatal or early postnatal β2AAs exposure could disrupt biochemical and morphological targets in multiple brain regions, leading to subsequent postnatal abnormalities in the brain development, causing nervous system damage and then neurodevelopment disorders [39, 40]. On the other hand, asthma, which was the indication mainly relevant in the present study, is characterized by shortness of breath, coughing or wheezing. This may result in interrupted or decreased oxygen supply that can lead to an impaired fetal oxygenation [41]. It is well known that impaired fetal oxygenation and hypoxia might have serious consequences on the development of brain and nervous system [42, 43]. Human studies have associated perinatal hypoxia with cerebral palsy [44].

While neurodevelopmental disorders in offspring were clearly attributed to β2AAs exposure in animal studies, it remains difficult to separate the effect of this medication from the effect of asthma in human studies [20, 24]. Therefore we extended the exposure window to 2 years prior to pregnancy and specified exposure periods: The association was mainly observed in female offspring whose mother used β2AAs both before and during pregnancy. As maternal β2AAs use both before and during pregnancy may suggest a higher severity of disease, the enhanced risk may be explained by the underlying disease, and also accompanied higher medication use. However, we did not observed a dose-response effect when we examined the cumulative dose of maternal β2AAs use during pregnancy. Thus, the association mainly observed for β2AAs use both before and during pregnancy was more likely be due to a cumulative effect over different exposure periods. Nevertheless, the evidence is not enough to disentangle the effect of a drug from its indication.

The association between maternal β2AAs use during pregnancy and an increased risk of CP in female offspring was only observed when analyses were restricted to term births. One potential explanation is that, in the present study, we did not have information on in-hospital use of β2AAs as a tocolytic agent, which means that maternal β2AAsuse for preterm pregnancies were misclassified as unexposed, thus attenuated the association when preterm births were included in the analyses.

Another consideration is that maternal use of β2AAs during pregnancy was associated with CP risk in female but not male offspring. In animal studies, sex difference in sensitivity to β2AAs was found in vitro experiment [45, 46]. In addition, men and women differ neurobiologically with respect to their response to brain injuries [47]. Our findings are partly supported by studies reporting that chronic maternal asthma was only associated with reduced growth in female fetuses, but not in male fetuses [48–51]. Although the results are preliminary, these findings suggested possible sex-selective mechanism in response to maternal asthma and the treatment during pregnancy.

This study has several strengths. The registry covers births of the entire country during the study period, and the linkage of several Danish population-based registers enabled us to estimate the effect of β2AAs exposure with almost complete follow-up, thus selection bias is less likely. Prescription data from the National Prescription Registry was used as proxy measures for drug usage. Since medical care is free of charge for residents in Denmark, the data is considered to be nearly complete and underreporting and recall bias are minimal. An additional strength is that CP cases in Danish National Cerebral Palsy Registry were diagnosed by strict inclusion criteria according to experts’ review of the children’s medical records, which reduces misclassification in disease diagnosis. Furthermore, the availability of data on potential confounding variables, such as sociodemographic factors, paternal diseases history, provides more options for confounder control.

Our findings should also be interpreted in light of the limitations. First, misclassification of the exposure should be taken into consideration. We assumed that women who filled a prescription of β2AAs also took the medication, which may not always be the case. In addition, the prescription registry didn’t include β2AAs administered during inpatient admission, which limits our ability to study maternal β2AAs used as a tocolytic agent because inpatient prescription is more common. Second, to be recorded as a CP case in the Danish National Cerebral Palsy Register, the child must survive to at least 1 year of age. Therefore, we may not include severe CP cases who died in pregnancy, at birth, or during early infancy. Thus, if there is a causal association between β2AAs use and CP, the observed association may be under ascertained. Third, in the present study, CP cases born after 2000 would not have a chance for receiving a validated CP diagnosis. Meanwhile, children exposed to β2AAs tended to be born in earlier calendar years. Therefore, more invalid CP cases born in years of lower β2AAs exposure could attenuate the association between β2AAs use and increased CP risk, if it exists. This hypothesis was supported by a stronger association observed in our further subanalyses restricted to children born before 1999. Finally, the small number of cases for some strata limits the statistical power, including the strata based on different exposure periods. Also, due to the limited number of cases, sibling analysis or stratified analysis by trimester of pregnancy or maternal use of glucocorticoids would not be informative. Therefore, we could not further test the robustness of our findings. However, when we restricted the main analyses in subgroup with no maternal use of glucocorticoids, there was a similar trend of increased CP risk in female offspring born at term who were exposed to maternal use of β2AAs, which suggested the observed association was less likely to be explained by the concurrent treatment of glucocorticoids.

In conclusion, we observed an association between maternal β2AAs used during pregnancy and an enhanced risk of CP in female offspring, but an effect of the underlying asthmatic disease could not be ruled out.

## Supporting information

S1 Table Association between maternal β2AAs usage and cerebral palsy in female offspring born at term with birth year of 1998–1999. (DOCX)

S2 Table Association between estimated cumulative dose of maternal β2AAs usage^a^ and cerebral palsy in female offspring born at term. (DOCX)

S3 Table Association between maternal β2AAs usage and cerebral palsy in female offspring born at term by specific drugs. (DOCX)

S4 Table Association between maternal β2AAs usage and cerebral palsy in female offspring born at term restricted to subgroup with no maternal use of glucocorticoids. (DOCX)
