# Supplementary material for: Risk of childhood cerebral palsy following prenatal exposure to ß2-adrenergic receptor agonist: A nationwide cohort study
Source: PLoS One. 2018 Aug 16;13(8):e0202078. doi: 10.1371/journal.pone.0202078 (PMC6095523; doi:10.1371/journal.pone.0202078)
Supplement: S1 Table — (DOCX) [file pone.0202078.s001.docx]

**S1 Table. Association between maternal β2AAs Usage and Cerebral Palsy in female Offspring born at term with birth year of 1998-1999**

| **Beta 2adrenoreceptor agonists use** | **Offspring without CP**  **(n=59874)** | **Offspring with CP**  **(n=82)** | **cOR(95%CI)** | **Model 1**  **aOR(95%CI)^a^** | **Model 2**  **aOR(95%CI)^b^** |
| --- | --- | --- | --- | --- | --- |
| **No use during pregnancy** | 56868(99.87) | 73(0.13) | Ref | Ref | Ref |
| **Use during pregnancy** | 3006(99.70) | 9(0.30) | 2.33(1.17,4.67)* | 2.34(1.16,4.69)* | 2.50(1.22,5.11)* |
|  |  |  |  |  |  |
| **Never use** | 48418(99.88) | 58(0.12) | - |  |  |
| **Use only before pregnancy** | 8450(99.82) | 15(0.18) | 1.48(0.84,2.62) | 1.69(0.92,3.08) | 1.71(0.94,3.13) |
| **Use only during pregnancy** | 1166(99.74) | 3(0.26) | 2.15(0.67,6.87) | 2.18(0.68,7.01) | 2.25(0.70,7.23) |
| **Use both before and during pregnancy** | 1840(99.67) | 1846(0.33) | 2.72(1.17,6.32)* | 2.86(1.22,6.71)* | 3.21(1.34,7.74)* |

^a^Adjusted for year of birth, parity, maternal age, paternal age, maternal cohabitation status, maternal education, maternal smoking, maternal history of cerebral palsy.

^b^Additionally adjusted for maternal history of hospital –diagnosed asthma based on model 1.

*p<0.05.
