# Supplementary material for: Risk of childhood cerebral palsy following prenatal exposure to ß2-adrenergic receptor agonist: A nationwide cohort study
Source: PLoS One. 2018 Aug 16;13(8):e0202078. doi: 10.1371/journal.pone.0202078 (PMC6095523; doi:10.1371/journal.pone.0202078)
Supplement: S2 Table — (DOCX) [file pone.0202078.s002.docx]

**S2 Table. Association between estimated cumulative dose of maternal β2AAs Usage^a^ and Cerebral Palsy in female Offspring born at term**

| **Beta 2adrenoreceptor agonists use** | **Offspring without CP**  **(n=205,503)** | **Offspring with CP**  **(n=250)** | **cOR(95%CI)** | **Model 1**  **aOR(95%CI)^b^** | **Model 2**  **aOR(95%CI)^c^** |
| --- | --- | --- | --- | --- | --- |
| **No use during pregnancy** | 196453(99.88) | 232(0.12) | Ref | Ref | Ref |
| ≤ **P_75_ of Cumulative dose** | 4861(99.75) | 12(0.25) | 2.09(1.17,3.74)* | 2.03(1.31,3.63)* | 2.12(1.71,3.84)* |
| **> P_75_ of Cumulative dose** | 4189(99.86) | 6(0.14) | 1.21(0.54,2.79) | 1.21(0.54,2.72) | 1.36(0.57,3.00) |

^a^Cumulative dose was estimated by multiplying number of packages redeemed for each prescription by number of defined daily doses in each package, and then summing across all prescriptions during pregnancy.

^b^Adjusted for year of birth, parity, maternal age, paternal age, maternal cohabitation status, maternal education, maternal smoking, maternal history of cerebral palsy.

^c^Additionally adjusted for maternal history of hospital –diagnosed asthma based on model 1.

*p<0.05.
