# Supplementary material for: Risk of childhood cerebral palsy following prenatal exposure to ß2-adrenergic receptor agonist: A nationwide cohort study
Source: PLoS One. 2018 Aug 16;13(8):e0202078. doi: 10.1371/journal.pone.0202078 (PMC6095523; doi:10.1371/journal.pone.0202078)
Supplement: S3 Table — (DOCX) [file pone.0202078.s003.docx]

**S3 Table. Association between maternal β2AAs Usage and Cerebral Palsy in female Offspring born at term by specific drugs**

| **Beta 2 adrenoreceptor agonists use** | **Offspring without CP** | **Offspring with CP** | **cOR(95%CI)** | **Model 1**  **aOR(95%CI)^a^** | **Model 2**  **aOR(95%CI)^b^** |
| --- | --- | --- | --- | --- | --- |
| ***Salbutamol*** |  |  |  |  |  |
| **No β2AAs use during pregnancy** | 196453(99.88) | 232(0.12) | Ref | Ref | Ref |
| **Use during pregnancy** | 2630(99.73) | 7(0.27) | 2.25(1.06,4.78)* | 2.22(1.04,4.71)* | 2.31(1.06,5.02)* |
|  |  |  |  |  |  |
| **Never use β2AAs** | 18141(99.88) | 210(0.12) | Ref | Ref | Ref |
| **Use only before pregnancy** | 15040(99.8) | 22(0.15) | 1.24(0.81,1.96) | 1.30(0.83,2.03) | 1.32(0.83,2.08) |
| **Use only during pregnancy** | 1062(99.81) | 2(0.19) | 1.63(0.40,6.56) | 1.60(0.39,6.45) | 1.63(0.40,6.56) |
| **Use both before and during pregnancy** | 1568(99.88) | 5(0.32) | 2.76(1.13,6.70)* | 2.73(1.12,6.65)* | 3.01(1.19,7.62)* |
|  |  |  |  |  |  |
| ***Terbutaline*** |  |  |  |  |  |
| **No β2AAs use during pregnancy** | 196453(99.88) | 232(0.12) | Ref | Ref | Ref |
| **Use during pregnancy** | 5306(99.81) | 10(0.19) | 1.59(0.85,3.01) | 1.58(0.84,2.98) | 1.82(0.96,3.46) |
|  |  |  |  |  |  |
| **Never use β2AAs** | 18141(99.88) | 210(0.12) | Ref | Ref | Ref |
| **Use only before pregnancy** | 15040(99.85) | 22(0.15) | 1.24(0.81,1.96) | 1.26(0.80,1.98) | 1.32(0.84,2.07) |
| **Use only during pregnancy** | 2703(99.82) | 5(0.18) | 1.60(0.65,3.88) | 1.58(0.65,3.85) | 1.69(0.70,4.12) |
| **Use both before and during pregnancy** | 2603(99.81) | 5(0.19) | 1.66(0.68,4.03) | 1.65(0.68,4.00) | 2.11(0.86,5.19) |

^a^Adjusted for year of birth, parity, maternal age, paternal age, maternal cohabitation status, maternal education, maternal smoking, maternal history of cerebral palsy.

^b^Additionally adjusted for maternal history of hospital –diagnosed asthma based on model 1.

*p<0.05.
