# Supplementary material for: Risk of childhood cerebral palsy following prenatal exposure to ß2-adrenergic receptor agonist: A nationwide cohort study
Source: PLoS One. 2018 Aug 16;13(8):e0202078. doi: 10.1371/journal.pone.0202078 (PMC6095523; doi:10.1371/journal.pone.0202078)
Supplement: S4 Table — (DOCX) [file pone.0202078.s004.docx]

**S4 Table. Association between maternal β2AAs Usage and Cerebral Palsy in female Offspring born at term restricted to subgroup with no maternal use of glucocorticoids**

| **Beta 2 adrenoreceptor agonists use** | **Offspring without CP** | **Offspring with CP** | **cOR(95%CI)** | **Model 1**  **aOR(95%CI)^a^** | **Model 2**  **aOR(95%CI)^b^** |
| --- | --- | --- | --- | --- | --- |
| **No use during pregnancy** | 195457(99.88) | 231(0.12) | Ref | Ref | Ref |
| **Use during pregnancy** | 6230(99.84) | 10(0.16) | 1.37(0.73,2.57) | 1.35(0.72,2.56) | 1.32(0.69.2.52) |
|  |  |  |  |  |  |
| **Never use** | 180952(99.88) | 209(0.12) | Ref | Ref | Ref |
| **Use only before pregnancy** | 14505(99.85) | 22(0.15) | 1.31(0.85,2.04) | 1.31(0.83,2.06) | 1.27(0.80,2.01) |
| **Use only during pregnancy** | 3264(99.85) | 5(0.15) | 1.33(0.55,3.22) | 1.31(0.54,3.20) | 1.24(0.51,3.04) |
| **Use both before and during pregnancy** | 2939(99.83) | 5(0.17) | 1.47(0.61,3.58) | 1.48(0.61,3.60) | 1.50(0.60,3.74) |

^a^Adjusted for year of birth, parity, maternal age, paternal age, maternal cohabitation status, maternal education, maternal smoking, maternal history of cerebral palsy.

^b^Additionally adjusted for maternal history of hospital –diagnosed asthma based on model 1.
